# EEG Markers as a Tool for the Individualization of Education and Optimization of Social Interventions for Children from Alcohol-Affected Families

**DOI:** 10.3390/brainsci16070769

**Published:** 2026-07-22

**Authors:** Małgorzata Chojak, Marta Czechowska-Bieluga

**Affiliations:** 1 Neuroeducation Research Lab, Faculty of Pedagogy, University of Marie Curie-Sklodowska, 20-031 Lublin, Poland; 2 Department of Social Pedagogy, Faculty of Pedagogy, University of Marie Curie-Sklodowska, 20-031 Lublin, Poland

**Keywords:** neuroeducation, EEG, children, alcohol-affected families, executive functions, emotional regulation, social intervention

## Abstract

**Highlights:**

**What are the main findings?**

- Children from alcohol-affected families exhibited a distinct neurofunctional profile characterized by impaired attention regulation, increased physiological arousal, and elevated somatic tension reflected in resting-state qEEG markers.
- Theta–Beta Ratio measures demonstrated the strongest discriminatory value among all analyzed qEEG markers, whereas frontal alpha asymmetry did not significantly differentiate the groups after correction for multiple comparisons.

**What are the implications of the main findings?**

- The proposed hierarchy of qEEG markers provides an evidence-based framework for prioritizing educational and social interventions according to children’s neurofunctional needs.
- Resting-state qEEG may serve as an objective complementary tool supporting individualized educational planning and multidisciplinary social support for children from alcohol-affected families.

**Abstract:**

**Background**: Children growing up in alcohol-affected families are exposed to chronic stress, adverse childhood experiences (ACEs), emotional insecurity, and environmental instability, all of which may influence neurodevelopmental processes. Numerous EEG markers have been proposed as indicators of attentional regulation, emotional functioning, and stress responsivity; however, their relative diagnostic and practical value remains unclear. The aim of the present study was to verify whether commonly reported EEG markers remain valid indicators of neurofunctional difficulties in children from alcohol-affected families, to establish their hierarchy of importance, and to determine how identified neurofunctional profiles may inform the sequencing of educational interventions and the development of individualized support plans used by educators and social workers. **Methods**: The study included children aged 6–10 years from alcohol-affected families (n = 20) and a control group from non-dysfunctional family environments (n = 25). Resting-state EEG recordings were conducted under eyes-open and eyes-closed conditions, with analyses focused on the eyes-open condition. Quantitative EEG (qEEG) indices included global, frontal, prefrontal, and midline Theta–Beta Ratio (TBR), frontal alpha asymmetry (FAA), temporal beta stress and parietal beta2 tension. EEG preprocessing was performed using EEGLAB and included artifact rejection, filtering, epoch segmentation, and spectral power analysis. Group differences were analyzed using Welch’s *t*-tests with Benjamini–Hochberg correction for multiple comparisons. **Results**: The analyzed EEG markers differed in their ability to distinguish children from alcohol-affected families and controls. The strongest effects were observed for Theta–Beta Ratio (TBR) measures, particularly in frontal and prefrontal regions, indicating impairments in attention regulation, executive functioning, and self-control. Elevated temporal beta stress and parietal beta2 tension reflected increased physiological arousal and chronic stress. In contrast, frontal alpha asymmetry (FAA), commonly associated with depressive emotional processing, was not significant after correction for multiple comparisons. The obtained findings enabled the establishment of a hierarchy of neurofunctional markers, with attentional and executive-function indicators demonstrating greater importance than markers related to depressive symptomatology. **Conclusions**: The EEG profile of children from alcohol-affected families is characterized primarily by chronic stress, heightened physiological activation, and impaired attention regulation rather than by neurophysiological patterns associated with depression. The results suggest that educational difficulties in this group may stem mainly from deficits in attention control, inhibitory processes, and cognitive flexibility. Consequently, educational interventions should prioritize learning strategies, attentional training, and self-regulated learning skills. The identified hierarchy of EEG markers may also support the development of individualized educational plans and social-support programs, including participation in structured extracurricular activities and interventions aimed at strengthening executive and learning-related competencies. However, given the pilot nature of the present study and the relatively small sample size, these findings should be considered preliminary. Replication in larger, more diverse, and independent cohorts is necessary to confirm the stability, reliability, and generalizability of the identified neurofunctional profile and the proposed hierarchy of qEEG markers before they can be recommended for broader educational and social applications.

## 1. Introduction

Children raised in alcohol-affected families constitute a high-risk group for the development of emotional, cognitive, and social difficulties. Contemporary research indicates that the consequences of growing up in an environment characterized by alcohol misuse are multifactorial and result from the interaction of genetic burden, chronic family stress, emotional instability, and caregiving neglect [1,2,3]. These children are more likely to exhibit behavioral disorders, impulsivity, difficulties in emotional regulation, symptoms of anxiety and depression, and an increased susceptibility to substance use during adolescence and adulthood [2,4]. Studies also emphasize the significance of an adverse family climate characterized by conflict, violence, unpredictability, and limited emotional support. These factors negatively affect children’s psychological development and may hinder the formation of secure interpersonal relationships [3,5]. Numerous analyses further indicate that children raised in alcohol-affected families more frequently experience feelings of insecurity, destabilized family relationships, and chronic emotional tension, all of which may influence their social, educational, and health-related functioning over the long term.

Researchers have also drawn attention to the cognitive functioning of children of parents with alcohol use disorders. Numerous studies have demonstrated reduced academic achievement and difficulties with attention, working memory, and executive control, which may translate into educational and social problems [1,4]. Increased rates of adaptive difficulties, problems with behavioral organization, reduced school motivation, and peer relationship difficulties have also been reported. At the same time, contemporary approaches increasingly move away from stereotypical portrayals of all children from alcohol-affected families as a homogeneous dysfunctional group. Greater emphasis is now placed on protective factors such as support from other adults, a stable school environment, and access to psychological assistance, all of which may substantially reduce the negative effects of growing up in an alcohol-affected family [2,5]. Consequently, current perspectives emphasize not only the elevated risk of mental health disorders and adaptive difficulties but also the considerable individual variability in developmental outcomes and the possibility of resilience among some children [3,4]. This suggests that children raised in alcohol-affected families do not constitute a homogeneous group in terms of psychosocial development and that their functioning depends on the interaction between risk and protective factors present within family, school, and broader social environments.

Such significant variability in the functioning of children from alcohol-affected families highlights the need to move beyond schematic support models toward approaches based on individualized assessment of children’s needs and resources. In practice, this requires consideration not only of observable emotional or educational difficulties but also of protective factors, adaptive mechanisms, and the specific characteristics of the family and school environment. The absence of in-depth assessment may lead to inaccurate identification of the sources of children’s problems, thereby reducing the effectiveness of educational and social interventions. Insufficient assessment of the needs of children from alcohol-affected families constitutes one of the main barriers to effective social and educational interventions. The literature emphasizes that the difficulties experienced by these children are often interpreted solely as behavioral problems or manifestations of school maladjustment, whereas their origins remain strongly associated with chronic family stress, emotional neglect, and the consequences of living in an environment affected by addiction [6,7]. The lack of comprehensive and multidimensional assessment leads to support strategies that are schematic and insufficiently tailored to the individual needs of the child.

The relationship between assessment and the possibility of personalizing support results from the fact that accurate recognition of a child’s psychosocial functioning enables the identification of actual deficits, resources, and protective factors, thereby allowing for the development of appropriate therapeutic, educational, and social interventions [6,8]. Research also indicates that children from alcohol-affected families often remain insufficiently “visible” to support systems because their difficulties may be masked by adaptive strategies such as social withdrawal, excessive self-reliance, or compensatory academic achievement [9]. Mismatched support interventions may reduce the effectiveness of both social services and school-based assistance. Contemporary interdisciplinary approaches assume that effective support requires the integration of psychological, educational, and environmental assessment, since children’s functioning results from the interaction of family, emotional, and educational factors [3,5]. Studies on support systems for children experiencing parental alcohol misuse indicate that schools and social institutions often have limited tools for identifying the problem, which hinders the implementation of individualized and long-term interventions [9,10,11]. Consequently, support efforts tend to focus primarily on reducing current behavioral difficulties rather than fostering emotional competencies, a sense of security, and psychological resilience. Research findings therefore underline the necessity of early and multidimensional assessment as a fundamental condition for effective individualization of educational and social interventions.

Contemporary approaches to the assessment of children raised in alcohol-affected families increasingly incorporate neurophysiological methods that enable a more objective evaluation of cognitive and emotional functioning. One method of particular importance in this context is electroencephalography (EEG), currently regarded as a relatively inexpensive, safe, and widely accessible technique for examining brain activity. EEG enables the recording of bioelectrical neuronal activity with high temporal resolution, making it possible to analyze cognitive and emotional processes in near real time. Unlike more expensive neuroimaging methods such as functional magnetic resonance imaging (fMRI) or positron emission tomography (PET), EEG is non-invasive, relatively brief, and suitable for use with younger children and individuals experiencing adaptive difficulties [12,13]. Furthermore, advances in mobile technologies and computerized signal analysis systems have made modern EEG equipment increasingly user-friendly, while the cost of conducting EEG examinations remains substantially lower than that of advanced neuroimaging techniques. At the same time, EEG meets high safety standards because it does not involve ionizing radiation and can be repeatedly used in longitudinal studies involving children and adolescents [14].

The literature emphasizes that EEG is increasingly applied in studies examining children exposed to chronic environmental stress, emotional neglect, and traumatic experiences. Analyses of brain waves and event-related potentials make it possible to identify differences related to attention, emotional regulation, impulsivity, and executive functioning [15,16]. Research indicates that children growing up in high psychosocial risk environments more frequently demonstrate alterations in theta and beta wave activity, which may be associated with attention difficulties, elevated emotional tension, and self-regulation impairments [17,18]. In the context of children raised in alcohol-affected families, EEG may therefore provide valuable information complementing traditional psychological and educational assessment methods and may support the process of individualizing educational and social interventions.

An important argument supporting the use of EEG lies in its capacity to identify neurophysiological markers associated with attention, executive control, emotional regulation, and stress reactivity. Research indicates that children raised in alcohol-affected or abusive families demonstrate characteristic patterns of neural activity associated with self-regulation difficulties and impaired adaptive functioning. Offspring of individuals with alcohol use disorders exhibit increased cortical excitability and weakened inhibitory control, which are associated with impulsivity, reduced behavioral control, and tendencies toward externalizing behaviors [19]. Studies employing event-related potentials (ERP), particularly NoGo-P3 paradigms, have demonstrated reduced amplitude and altered topography of neural responses related to response inhibition, suggesting deficits in executive control and inhibitory mechanisms [20]. Further analyses also indicate broader motivational–executive dysfunctions and increased vulnerability to alcohol use disorders [21].

Additional evidence highlights the role of neurophysiological dysregulation in other domains of functioning. Sleep disturbances have been identified as early indicators of developmental risk and may precede the onset of substance-related behaviors [22]. Studies involving children exposed to psychological maltreatment demonstrate impairments in emotional processing and regulation reflected in altered neural responses to affective stimuli. Differences in frontal alpha asymmetry (FAA) have also been observed, distinguishing more resilient individuals from those demonstrating maladaptive functioning patterns [23]. Longitudinal findings further indicate that early trauma exposure is associated with later behavioral problems, emotional disorders, and increased risk of substance use [24].

Another important area of investigation concerns the long-term consequences of the absence of early intervention for children raised in alcohol-affected families. Longitudinal studies indicate an elevated risk of developing substance use disorders, anxiety disorders, and depression during adolescence and adulthood [25]. The Adverse Childhood Experiences (ACE) framework demonstrates a strong relationship between the number of adverse childhood experiences and the risk of mental and somatic disorders later in life [24,26]. Parental alcohol misuse has also been associated with difficulties in school adaptation, lower academic achievement, and reduced educational attainment in adulthood. Individuals raised in such environments more frequently experience difficulties in establishing stable interpersonal relationships and maintaining satisfying social bonds [27]. Research on childhood maltreatment and neglect additionally points to increased risks of depression, anxiety disorders, post-traumatic stress disorder, substance use disorders, and poorer physical health outcomes [28], thereby contributing to the persistence of social and health inequalities in adulthood [29].

From a neurodevelopmental perspective, the absence of early and targeted intervention may lead to the consolidation of maladaptive neural activity patterns. This process can be explained through mechanisms of experience-dependent plasticity, according to which repeated emotional states and behaviors strengthen corresponding neural pathways. At the same time, the brain’s capacity for neural reorganization decreases with age, as described within the framework of sensitive periods [30,31]. This means that the absence of timely support may result in the long-term persistence of difficulties related to attention, emotional regulation, behavioral control, and social functioning.

Research indicates that appropriately timed and multicomponent interventions—including relational support, executive-function training, self-regulation development, and educational interventions—may partially reverse unfavorable neurophysiological changes and improve long-term developmental outcomes [18,32]. Importantly, contemporary intervention research consistently suggests that the effectiveness of support increases when priority is given to the areas of greatest functional difficulty experienced by the child. Interventions targeting the most pronounced deficits, particularly in attention regulation, executive functioning, and self-regulation, tend to produce broader developmental benefits and facilitate subsequent improvements in emotional, social, and educational functioning. This assumption is consistent with developmental models emphasizing the foundational role of executive functions in learning and adaptation [33]. Research conducted within the framework of developmental neuroscience further indicates that strengthening core self-regulatory processes creates conditions for subsequent improvements in academic achievement, emotional regulation, and social competence [34]. Although different intervention models emphasize various mechanisms of change, the available evidence generally supports a hierarchical approach in which support is first directed toward the most limiting developmental difficulties before addressing secondary consequences [35].

The literature further indicates that support for children exposed to chronic family adversity is most effective when it is delivered through coordinated actions involving multiple institutions, including schools, social services, healthcare professionals, and psychological support systems. Ecological and developmental models consistently emphasize that children’s functioning results from interactions between family, educational, community, and broader social systems [36]. Consequently, multidisciplinary and interagency approaches have been associated with better educational, social, and mental health outcomes than isolated interventions conducted within a single service sector [37]. Effective collaboration, however, requires that all professionals involved in the support process operate on the basis of the same diagnostic information and a shared understanding of the child’s strengths, difficulties, and developmental needs. Consequently, the identification of objective and comparable indicators of functioning becomes particularly important for planning and coordinating individualized interventions.

In this context, EEG markers may constitute a valuable complementary source of information supporting educational and social decision-making. By providing objective indicators of attention regulation, executive control, emotional processing, and stress reactivity, EEG-based assessment may facilitate the identification of priority intervention targets and support the development of coordinated, evidence-based support plans implemented across educational, social, and healthcare systems.

Although numerous studies have examined EEG correlates of adverse childhood experiences and parental alcohol misuse, most have focused on isolated neurophysiological measures or specific cognitive functions. Consequently, relatively little attention has been devoted to comparing the practical utility of different qEEG markers within a single analytical framework or to translating neurophysiological findings into recommendations for educational and social practice. The present study addresses this gap by systematically evaluating multiple qEEG indices, establishing their relative diagnostic value, and proposing a hierarchy of neurofunctional markers that can guide the prioritization of individualized educational and social interventions. This translational perspective extends current knowledge beyond the description of EEG abnormalities and provides a practical framework for integrating neurophysiological evidence into interdisciplinary support for children from alcohol-affected families.

## 2. Materials and Methods

### 2.1. Participants and Recruitment

The study was conducted in October and November 2022 among primary school children in Lublin city in Poland. Participants were recruited in cooperation with school pedagogues, who distributed study invitations to parents and legal guardians. Caregivers who expressed interest in participation received detailed information about the study procedures and provided written informed consent prior to enrollment. Parents additionally completed a health questionnaire designed to exclude children with neurological disorders, developmental conditions, or other medical factors that could potentially affect EEG recordings.

Information regarding alcohol-related problems within the family was obtained during the screening process based on documentation provided by school staff familiar with the family situation. In the Polish educational system, school pedagogues are members of interdisciplinary teams responsible for planning and coordinating support for children at risk of social maladjustment and educational difficulties. In accordance with national regulations, assessment of a child’s family environment and psychosocial situation constitutes a mandatory element of the support process and is based on information collected from multiple sources, including teachers, psychologists, social services, parents, and educational documentation. Consequently, school pedagogues possess reliable knowledge regarding family functioning, supported by standardized assessment procedures and interdisciplinary case evaluation. The information was used solely for participant classification and group assignment and did not involve independent clinical diagnosis of parental alcohol use disorders.

The final sample consisted of two groups of children aged 6–10 years: a clinical group (n = 20) comprising children from alcohol-affected families and a control group (n = 25) including children from non-dysfunctional family environments. In the clinical group, the sex distribution was balanced (50% girls and 50% boys), with a mean age of 8.50 years. In the control group, the mean age was 7.84 years, with a slightly higher proportion of boys (52%) than girls (48%). The sample size reflects the specific challenges associated with recruiting children from alcohol-affected families. Obtaining parental consent is often difficult due to the sensitive nature of alcohol-related family problems and concerns regarding disclosure of family circumstances. Recruitment was additionally affected by the COVID-19 peri-pandemic period, during which participation in face-to-face assessments was limited by health-related concerns. Given these constraints, the study was designed as a pilot investigation aimed at the preliminary identification and verification of neurophysiological markers characteristic of children from alcohol-affected families. The findings provide a basis for future studies involving larger samples.

### 2.2. Procedure

The experimental procedure consisted of two resting-state EEG conditions conducted in a quiet and dimly lit laboratory room. The examinations were carried out in the presence of a parent or legal guardian in order to enhance the participants’ sense of safety and reduce emotional tension that could affect the recording procedure. Prior to the examination, children received standardized instructions and were familiarized with the course of the procedure to reduce stress and minimize movement-related artifacts. Participants were seated comfortably in an upright position approximately 1 m from a neutral fixation point positioned at eye level.

Two resting-state recording conditions were administered in a fixed order:

1. Eyes open (EO)—participants were instructed to remain still, minimize blinking and body movements, and focus their gaze on a fixed point for 3 min.
2. Eyes closed (EC)—participants remained relaxed with their eyes closed for 3 min while avoiding excessive movements and falling asleep.

For the purposes of the present analyses, only data from the eyes-open condition were used. The eyes-open resting-state paradigm was selected because it better reflects the neurofunctional state associated with sustained attention, environmental monitoring, and cognitive readiness, which are directly related to the executive and attentional processes examined in the present study. Moreover, the eyes-open condition reduces the influence of dominant occipital alpha activity typically observed during eyes-closed recordings, thereby facilitating the interpretation of frontal and frontocentral qEEG markers such as Theta–Beta Ratio and frontal alpha asymmetry. This approach also improves comparability with previous developmental studies investigating attentional regulation and executive functioning.

During EEG acquisition, several contextual and physiological variables were documented, including body position, time of day, medication use, sleep quality on the night preceding the examination, handedness, and the presence of observable behavioral artifacts (e.g., excessive movements, muscle tension, or signs of drowsiness). These factors were monitored to improve data quality and support the interpretation of individual variability.

EEG data were recorded using the **Mitsar EEG system** (Mitsar Co., Ltd., St. Petersburg, Russia) and **DataStudio software v1.11.0.659** (Mitsar Co., Ltd., St. Petersburg, Russia). EEG activity was recorded using a 19-channel system based on the international 10–20 electrode placement standard (Fp1, Fp2, F3, F4, F7, F8, Fz, C3, C4, Cz, T3/T4, T5/T6, P3, P4, Pz, O1, O2). Reference electrodes were placed at the mastoids (A1/A2). Electrode impedance was continuously monitored and maintained below 5–10 kΩ throughout the recording session.

EEG signals were acquired at a sampling rate of 1000 Hz. Before preprocessing and spectral analyses, all recordings were resampled offline to 500 Hz in EEGLAB to standardize the sampling frequency across all datasets and reduce computational load. All subsequent quantitative analyses were therefore performed on data sampled at 500 Hz.

### 2.3. EEG Measures (Functional Markers)

The study employed a set of quantitative EEG (qEEG) indicators reflecting neurofunctional processes associated with attention, executive control, emotional regulation, and somatic tension. The selection of metrics was integrative and based on established approaches in clinical, neuropsychological, and neurofeedback research.

The classification of these indices as biomarkers follows the BEST (Biomarkers, Endpoints, and other Tools) framework [38], which requires clear definition, validation, and specification of functional roles.

The following markers were analyzed:

(1) Theta–Beta Ratio (TBR)

Defined as θ/(β1 + β2), where θ = 4–7 Hz and β = 13–30 Hz. Elevated TBR is associated with attention deficits and reduced executive control, particularly in fronto-central regions [34,35].

(2) Frontal Alpha Asymmetry (FAA)

Calculated as ln(α right) − ln(α left), typically for Fp2–Fp1 or F4–F3 electrode pairs. Negative values are associated with withdrawal tendencies and risk for internalizing problems, whereas positive values reflect approach-related motivation [39].

(3) Temporal Beta Stress

Defined as mean beta-band power (13–35 Hz) in temporal regions (T3, T4). Increased values may reflect heightened emotional tension, although partial contamination by muscle activity should be considered [40,41].

(4) Parietal EMG Tension Index

Based on beta2 power (20–35 Hz) in parietal regions (P3, P4, Pz), reflecting somatic tension and muscular activation [41].

A summary of the computational framework is presented in Table 1.

### 2.4. Data Processing

EEG preprocessing and analysis were conducted using EEGLAB implemented in the MATLAB R2025b environment. EEG signals were imported in European Data Format (EDF/BDF), and each dataset was linked to a metadata file containing demographic, clinical, and recording-related information, including participant age, sex, handedness, recording condition, and quality-control annotations.

The preprocessing pipeline included:

- channel standardization according to the international 10–20 system;
- visual inspection of raw EEG recordings;
- removal of movement, ocular, and muscle artifacts;
- high-pass filtering (0.5–1 Hz) and low-pass filtering (40–45 Hz);
- application of a 50 Hz notch filter to reduce electrical line noise;
- segmentation into fixed 2 s epochs;
- rejection of artifact-contaminated epochs;
- selection of ≥120–180 s of artifact-free data per condition (at least 120 s of artifact-free EEG was retained for each participant; when available, up to 180 s of clean data were included).

EEGLAB functions were additionally used for signal inspection, artifact rejection, epoch management, and spectral decomposition. Artifact detection was supported by both automated threshold-based procedures and manual visual verification performed by trained researchers. Ocular and movement artifacts were first identified using automatic amplitude (±100 µV) and abnormal trend criteria, followed by independent visual inspection by two experienced EEG analysts. Independent component analysis (ICA) was not applied because, given the characteristics of the present dataset (short resting-state recordings and a 19-channel montage), visual inspection combined with epoch rejection was considered a more conservative and appropriate artifact-removal strategy.

Absolute and relative power values were computed for the following frequency bands:

- delta (1–3 Hz);
- theta (4–7 Hz);
- alpha (8–12 Hz);
- beta1 (13–20 Hz);
- beta2 (21–30 Hz).

Spectral power was estimated using the Welch method (2 s Hamming window, 50% overlap). Relative power was expressed as the proportion of total power within the 1–40 Hz range.

Spectral power was estimated using Welch’s method (2 s Hamming window, 50% overlap). Relative power was expressed as the proportion of total power within the 1–40 Hz range. Power spectral density (PSD) was calculated using Welch’s estimator according to Equation (1):

(1) $$P_{xx}\left(f\right)=\frac{1}{LU}\sum_{l=0}^{L-1}{\left|\sum_{n=0}^{N-1}w\left(n\right)x_{l}\left(n\right)e^{-j2pifn}\right|}^{2}$$

where is the number of overlapping segments, *N* is the segment length, *w*(*n*)*w*(*n*)*w*(*n*) denotes the Hamming window, *U* is the window normalization factor, and *xl*(*n*)*x_l*(*n*)*xl* (*n*) is the lll-th signal segment.

Power spectral density (PSD) was calculated using Welch’s estimator: *Pxx*(*f*) = 1/K Σ|FFT(*w*(*n*)*x_k*(*n*))|^2^/(*U·Fs*), where K is the number of overlapping segments, *w* is the Hamming window, *U* is the window normalization factor, and *Fs* is the sampling frequency. Absolute band power was obtained by integrating the PSD within each frequency band, whereas relative power was calculated as the ratio of band power to the total power in the 1–40 Hz range.

All analysis scripts, preprocessing parameters, and quality-control logs were archived in the project repository with full anonymization of participant data. Information regarding alcohol-related problems within the family was available only to professionals directly involved in the recruitment process and was not disclosed to researchers responsible for EEG data collection or analysis. Participant identification codes were used throughout the study, and the research database contained no information enabling the direct identification of children or their families.

To minimize the risk of stigmatization, recruitment procedures did not explicitly refer to parental alcohol misuse. Both children and their caregivers were informed that the study concerned child development and learning processes. Written informed consent was obtained from parents or legal guardians prior to participation. In addition, immediately before the assessment, each child received age-appropriate information about the purpose and procedures of the study and was asked to provide assent to participate. Children were also informed that they could withdraw from the study at any time without providing a reason and without any negative consequences. All procedures were conducted in accordance with applicable ethical standards for research involving minors and the handling of sensitive family-related information.

### 2.5. Statistical Analysis

Prior to the main analyses, the distribution of the analyzed variables was assessed. Normality of distribution was verified using the Shapiro–Wilk test, which is recommended for small sample sizes. Additionally, skewness and kurtosis values as well as Q–Q plots were examined. Homogeneity of variances between groups was assessed using Levene’s test.

Differences between the clinical and control groups were analyzed using the independent-samples Student’s *t*-test. Statistical significance was set at *p* < 0.05. Effect size was estimated using Pearson’s *r* coefficient calculated from the *t* statistic according to the following formula: *r* = √[*t*^2^/(*t*^2^ + *df*)]. Values of *r* around 0.10 were interpreted as small effects, values around 0.30 as moderate effects, and values ≥ 0.50 as large effects.

Statistical power analysis was conducted using G*Power 3.1 for a two-tailed independent-samples *t*-test with α = 0.05 and statistical power of 1 − β = 0.80. Due to the pilot nature of the study and the sample size of N = 45, the analysis was treated as an assessment of the adequacy of the sample primarily for detecting moderate and large effects.

**Figure 1:**
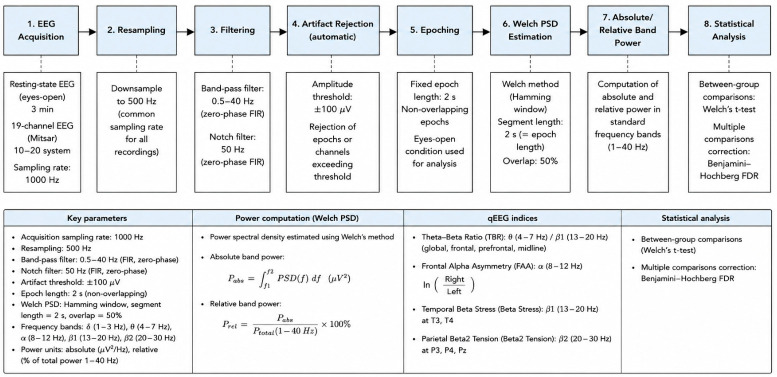
EEG data processing pipeline.

A schematic overview of the complete data-processing workflow is presented in Figure 1.

## 3. Results

### 3.1. Between-Group Differences in EEG Markers

Analyses were conducted to examine differences between the clinical and control groups in key EEG markers reflecting attention, executive control, motivation, emotional processing, stress reactivity, and somatic tension.

Table 2 presents descriptive statistics for the analyzed variables (means and standard deviations), results of Welch’s *t*-tests applied due to potential inequality of variances between groups, *p*-values adjusted using the Benjamini–Hochberg (BH) procedure to control for multiple comparisons, and effect sizes expressed as Cohen’s *d*.

The analysis of between-group differences revealed statistically significant differences between children from alcohol-affected families and children from the control group across most of the analyzed EEG markers (see Table 3).

The strongest effects were observed for Theta–Beta Ratio indices. Both Frontal TBR (*d* = 2.77, *p*adj < 0.001) and Global TBR (*d* = 2.68, *p*adj < 0.001) demonstrated very large effect sizes. A similar pattern was found for the Prefrontal Theta Beta1 index (*d* = 2.52, *p*adj < 0.001) and Motivation TBR midline (*d* = 1.35, *p*adj < 0.001). These findings suggest elevated theta activity relative to beta activity within frontal and prefrontal regions, potentially reflecting difficulties in attentional control, cognitive regulation, and executive functioning.

Children from alcohol-affected families also exhibited higher values of markers associated with stress and somatic tension. Temporal beta stress reached statistical significance with a large effect size (*d* = 1.06, *p*adj = 0.002), while Parietal beta2 tension also significantly differentiated the groups (*d* = 0.87, *p*adj = 0.009). These results may indicate increased emotional arousal as well as elevated muscular and physiological tension in the clinical group.

Regarding alpha activity, significantly lower values of Alpha Fp1 were observed in the clinical group compared with the control group (*d* = −0.93, *p*adj = 0.005). This finding may reflect altered frontal activation patterns associated with emotional regulation and motivational processes. For Alpha Fp2, only a statistical trend was observed (*p*adj = 0.074). No significant between-group differences were found for the frontal alpha asymmetry index FAA ln (*p*adj = 0.199).

### 3.2. EEG Profile of Children from Alcohol-Affected Families

The pattern of results allows the identification of a consistent neurofunctional profile distinguishing children from alcohol-affected families from controls.

This profile is characterized by:

- elevated TBR indices (global, frontal, prefrontal, and midline), indicating deficits in attentional control and executive functioning;
- elevated beta2 power in parietal regions, suggesting increased somatic and muscular tension;
- reduced alpha power in the prefrontal region (Fp1), associated with difficulties in emotional regulation;
- increased midline TBR, indicating reduced motivational engagement and task persistence.

**Figure 2:**
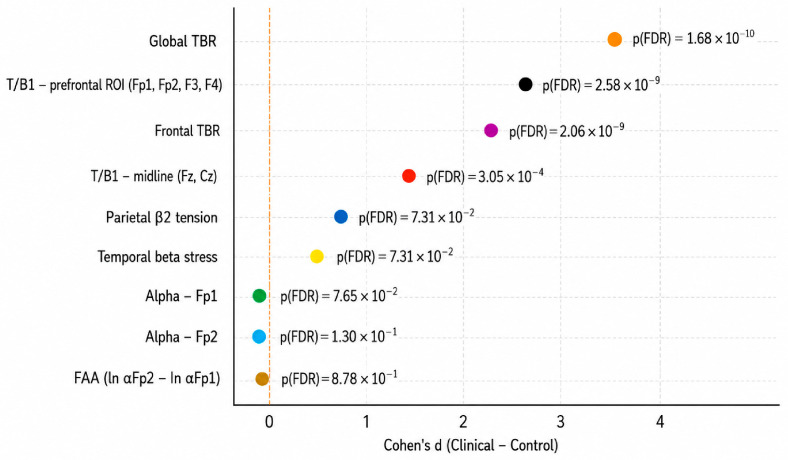
Summary of group differences.

These findings are illustrated in Figure 2.

## 4. Discussion

The present study indicates that children from alcohol-affected families exhibit a distinct neurofunctional profile characterized by elevated indices of attentional dysregulation, increased stress-related activation, heightened somatic tension, and altered frontal activity associated with emotional regulation. These findings should be interpreted within the broader framework of adverse childhood experiences (ACEs), family dysfunction, developmental trauma, and neurodevelopment under chronic stress. Parental alcohol misuse is not only an isolated family risk factor but also often co-occurs with emotional unpredictability, inconsistent caregiving, neglect, conflict, reduced parental availability, and chronic psychosocial stress. In this context, the child’s neurophysiological functioning may reflect adaptation to a persistently unstable developmental environment [34,42].

The elevated Theta–Beta Ratio (TBR) indices observed across global, frontal, and prefrontal regions suggest reduced attentional control, lower efficiency of executive regulation, and difficulties in maintaining stable cognitive engagement. This pattern is consistent with literature linking higher TBR values with attentional problems and weaker executive functioning, particularly in children with regulatory and developmental difficulties [43,44]. From the perspective of ACEs research, such findings may be interpreted as neurofunctional correlates of chronic exposure to adversity. Systematic reviews indicate that adverse childhood experiences are associated with executive function difficulties in children, including poorer inhibition, working memory, cognitive flexibility, and self-regulation [45]. These mechanisms play an important role in learning, school adaptation, emotional regulation, and social functioning. Therefore, elevated frontal and prefrontal TBR observed in the clinical group may represent neurofunctional correlates associated with reduced attentional regulation and executive control. However, these findings should not be interpreted as direct evidence of impaired educational functioning, but rather as objective indicators that may complement psychological and educational assessment.

An important contribution of the present study is the verification and hierarchical organization of EEG markers previously associated with children exposed to chronic family adversity. The strongest effects were observed for TBR measures, particularly within frontal and prefrontal regions, followed by temporal beta stress and parietal beta2 tension. In contrast, frontal alpha asymmetry did not significantly differentiate the groups. This pattern suggests that attentional regulation and executive control may constitute particularly relevant neurofunctional domains in this population, whereas markers traditionally linked to depressive emotional processing appear to demonstrate lower discriminatory value within the present sample. Although these findings should be interpreted with caution given the pilot nature of the study, they provide preliminary evidence that may help guide future research and inform the development of individualized educational and social support strategies based on the relative importance of different qEEG markers.

The family environment of children affected by parental alcohol problems may act as a source of chronic toxic stress. Toxic stress differs from temporary stress because it involves prolonged activation of stress-response systems without adequate buffering by stable and responsive caregiving. This type of exposure has been shown to affect brain systems involved in emotion regulation, executive control, threat detection, and physiological arousal [34,46,47]. The present findings are consistent with this model. Increased temporal beta stress and parietal beta2 tension suggest heightened emotional arousal, somatic tension, and physiological readiness to respond to threat. In children from alcohol-affected families, such a pattern may reflect hypervigilance, increased sensitivity to environmental cues, and difficulty returning to a calm baseline state after stimulation.

The observed elevation of stress-related EEG markers is particularly important for social intervention. Children raised in alcohol-affected families may not always present overt behavioral problems, yet their neurophysiological profile may indicate increased internal tension and reduced capacity for self-regulation. This supports the need for early, proactive, and trauma-informed social support rather than interventions limited solely to crisis situations. Effective social intervention should include systematic identification of family risk, cooperation between schools, social services, psychological support providers, and family assistants, as well as long-term monitoring of the child’s functioning [48].

Although lower alpha activity in the Fp1 region was observed in the clinical group, frontal alpha asymmetry did not significantly differentiate the groups. This finding suggests that attentional and executive-regulatory mechanisms may be more strongly associated with the functioning of children from alcohol-affected families than neurophysiological markers traditionally linked to depressive emotional processing. The lack of significant FAA effects may additionally reflect the developmental variability of frontal asymmetry patterns during middle childhood.

The results also correspond with research on children of parents with alcohol use problems. Studies indicate that offspring of alcohol-affected families are at increased risk of emotional, behavioral, motivational, and executive functioning difficulties. Nattala et al. [49] demonstrated that children of fathers with alcohol dependence were at risk for executive function impairments strongly associated with emotional and behavioral problems. Similarly, Simonič and Osewska [50] emphasized that growing up in an alcohol-affected family may result in long-term disturbances in emotional regulation, interpersonal trust, and coping with stress. Therefore, the EEG markers identified in the present study may reflect early neurofunctional correlates of difficulties that can later manifest as problems in learning, relationships, emotional stability, and social adaptation.

From an educational perspective, the present findings suggest the potential value of individualized learning environments for children from alcohol-affected families. Elevated TBR and stress-related markers may indicate neurofunctional characteristics associated with attentional regulation and physiological arousal, suggesting that some children could benefit from more structured, predictable, and regulation-supportive educational conditions. Such individualization may extend beyond the adaptation of academic content to include consideration of cognitive load, classroom stimulation, task duration, feedback style, and emotional safety. Potential educational strategies include shorter and clearly structured tasks, step-by-step instructions, frequent comprehension checks, reduced environmental distraction, predictable routines, visual schedules, additional time for task completion, and opportunities for movement or brief self-regulation breaks. Given that executive-control processes may be less efficient in some children, educational support may also involve external scaffolding of planning, organization, inhibitory control, and working memory, rather than relying exclusively on independent management of complex school demands [48].

The identified hierarchy of EEG markers suggests that interventions may benefit from placing particular emphasis on attentional regulation, executive functioning, and self-regulatory skills, as these domains demonstrated the strongest differentiation between groups. Emotional support and stress-reduction strategies remain important; however, the present findings suggest that difficulties related to attention control, cognitive organization, and executive regulation may represent important factors influencing educational participation. Consequently, educational programs may benefit from incorporating evidence-based approaches supporting learning strategies, attentional control, metacognitive skills, and executive functioning while simultaneously providing emotionally supportive and predictable learning environments.

In the area of social intervention, the findings highlight the potential value of integrated, multidimensional support. Intervention approaches may combine child-focused, family-focused, and school-based components. At the child level, support may address attentional regulation, executive functioning, self-regulation, emotional control, and stress reduction. At the family level, intervention may include parenting support, psychoeducation concerning the developmental impact of alcohol-related dysfunction, strengthening predictable caregiving routines, and referral to addiction treatment when appropriate. At the school level, teachers and pedagogues may benefit from training in recognizing signs of chronic stress and family adversity, avoiding punitive responses to dysregulated behavior, and implementing supportive educational accommodations.

The findings also have potential implications for social work practice. Unlike educational institutions, social services may directly address family-level factors contributing to developmental risk. Within the Polish system of social assistance, support activities may be formalized through individualized assistance plans, family support programs, and social contracts developed in cooperation with caregivers. These mechanisms may facilitate children’s participation in interventions targeting their most pronounced areas of difficulty, including executive-function training, structured extracurricular activities, specialist educational support, and programs aimed at strengthening self-regulation and learning competencies. The hierarchy of EEG markers identified in the present study may therefore help inform the prioritization of support activities and contribute to a shared evidence base for coordinated actions undertaken by schools, psychologists, social workers, and family support services.

The present findings may contribute to the development of practical guidelines for educational and social services. First, when appropriate, the assessment of children from alcohol-affected families may benefit from considering not only behavioral symptoms but also attention, executive functioning, emotional regulation, stress reactivity, and somatic tension. Second, school-based support may include regular monitoring by school pedagogues and psychologists, particularly during periods of family crisis or academic difficulty. Third, intervention plans may benefit from being individualized and based on the child’s functional profile rather than on family risk status alone. Fourth, cooperation between educational institutions and social services may be strengthened through systematic, confidential, and developmentally oriented collaboration that minimizes the risk of stigmatization. Fifth, the hierarchy of identified neurofunctional characteristics may help inform the prioritization of support activities, with particular attention to attentional regulation and executive functioning. Finally, future studies could evaluate intervention outcomes longitudinally using indicators such as improved attention, reduced emotional reactivity, greater task persistence, improved classroom participation, and reduced physiological signs of tension.

Overall, the present study adds to the growing body of evidence suggesting that children from alcohol-affected families may exhibit measurable neurofunctional differences even at the level of resting-state EEG activity. These findings are consistent with the broader literature on adverse childhood experiences (ACEs), family dysfunction, developmental trauma, and neurodevelopment under chronic stress. The results further suggest that qEEG markers may provide complementary information that could support the individualization of educational approaches and the planning of social interventions. However, given the pilot nature of the study, these findings should be regarded as preliminary and require replication in larger and more diverse samples before broader practical application can be considered. Importantly, EEG markers should not be interpreted as diagnostic indicators or used in isolation, but rather as complementary sources of information that may support comprehensive psychological, educational, and social assessment, thereby contributing to a more individualized understanding of children’s developmental and regulatory needs.

## 5. Conclusions

The present study demonstrated that children from alcohol-affected families exhibit a distinct neurofunctional profile characterized by elevated indices of attentional dysregulation, executive control difficulties, increased stress reactivity, heightened somatic tension, and altered patterns of emotional regulation observable in resting-state EEG activity. The strongest differences between the clinical and control groups were identified in markers associated with Theta–Beta Ratio, stress-related beta activity, and SMR-related regulation processes, suggesting that chronic exposure to dysfunctional family environments may be associated with measurable neurophysiological alterations during development.

The findings support contemporary models of adverse childhood experiences (ACEs), developmental trauma, and neurodevelopment under chronic stress, indicating that prolonged exposure to family dysfunction and parental alcohol-related problems may influence mechanisms responsible for cognitive regulation, emotional processing, and psychophysiological adaptation. Importantly, the observed EEG differences should not be interpreted exclusively as markers of pathology, but rather as indicators of developmental adaptation to persistent environmental stress and emotional instability.

From a practical perspective, the results highlight the potential value of EEG-derived markers as supportive tools for the individualization of educational and social interventions. Elevated TBR indices and stress-related activation patterns suggest that children from alcohol-affected families may require structured, predictable, and regulation-supportive educational environments, as well as interventions targeting attention, executive functioning, emotional regulation, and stress reduction. Trauma-informed and multidimensional approaches integrating educational, psychological, and social support may therefore be particularly important in this population.

The study additionally emphasizes the importance of early identification and interdisciplinary cooperation between schools, psychologists, social workers, and family support services. Educational and social interventions should be based not only on observable behavioral symptoms but also on the child’s broader functional and regulatory profile. EEG markers may provide complementary information supporting more individualized and developmentally sensitive intervention planning.

The study additionally emphasizes the importance of early identification and interdisciplinary cooperation among schools, psychologists, social workers, and family support services. Previous research indicates that interventions are most effective when multiple institutions coordinate their activities using a shared understanding of the child’s needs. Consequently, educational and social interventions should be based not only on observable behavioral symptoms but also on objective indicators of functional regulation.

The identified hierarchy of EEG markers may support the development of coordinated intervention plans in which different institutions address complementary aspects of the child’s functioning. While schools and educational specialists may focus primarily on strengthening attention regulation, executive functioning, learning strategies, and self-regulatory skills, social workers and family support services may address environmental and family-related factors that influence the child’s developmental outcomes. In the Polish system of social assistance, social workers may formalize support activities through individualized assistance plans and social contracts developed in cooperation with families. This creates opportunities to incorporate evidence-based recommendations into coordinated support strategies and to facilitate children’s participation in interventions targeting their most pronounced developmental difficulties, such as executive-function training, structured extracurricular activities, or specialized educational support.

The use of shared assessment data may therefore improve the coherence, continuity, and effectiveness of support provided across educational, psychological, and social-service systems. EEG markers may provide complementary information supporting more individualized, evidence-based, and developmentally sensitive intervention planning.

## 6. Limitations

Several limitations of the present study should be acknowledged. First, the relatively small sample size and exploratory character of the study limit the generalizability of the findings. Although significant differences and large effect sizes were identified for several EEG markers, replication in larger and more diverse samples is necessary to confirm the stability of the observed neurofunctional patterns.

Second, the study was based primarily on resting-state EEG measures and did not include additional behavioral, educational, neuropsychological, or multi-informant assessments that would allow direct verification of the functional significance of the observed neurophysiological differences. Consequently, the identified EEG alterations should be interpreted as potential neurofunctional correlates of cognitive and emotional regulation rather than as direct indicators of functional impairment or everyday educational, emotional, or social functioning. Although previous research has linked several of the analyzed qEEG markers with attentional regulation, executive functioning, and stress responsivity, the present study cannot determine the extent to which these neurophysiological characteristics translate into observable behavioral outcomes in individual children. Moreover, the practical application of EEG-based assessment should be viewed as complementary rather than alternative to comprehensive psychological, educational, and social evaluation. qEEG markers may provide objective information about neurofunctional processes and support the identification of children who may benefit from further assessment; however, they cannot replace multidisciplinary clinical judgment or standardized psychological and educational assessment. Future studies should therefore integrate EEG recordings with standardized behavioral, neuropsychological, educational, and psychosocial assessments to clarify the relationship between neurophysiological markers and real-world functioning and to improve the ecological validity of EEG-based assessment.

Third, although recording conditions were standardized, resting-state EEG activity may be influenced by contextual and physiological factors such as sleep quality, emotional state, fatigue, or recent stress exposure. While selected variables were monitored during data collection, future research should include broader control of potential covariates and longitudinal assessment designs.

Finally, the cross-sectional nature of the study does not allow causal interpretation of the observed relationships. Further longitudinal and multimodal studies are needed to better understand the developmental mechanisms linking chronic family adversity, stress exposure, and neurofunctional regulation in children from alcohol-affected families.

Future research should also examine the extent to which EEG-derived markers may predict educational functioning, emotional adaptation, social competence, and responsiveness to intervention over time. Particularly valuable would be studies integrating neurophysiological assessment with measures of executive functioning, school achievement, emotional regulation, and adverse childhood experiences (ACEs). Further investigations should additionally evaluate the effectiveness of individualized educational and psychosocial interventions designed for children from alcohol-affected families, including trauma-informed educational practices, executive-function training, stress-regulation interventions, and neurofeedback-based approaches. Such research could contribute to the development of more precise and evidence-based models of educational and social support for children exposed to chronic family adversity.

This study has several limitations that should be considered when interpreting the findings.

## Figures and Tables

**Table 1:** Computational framework used in the present study.

Metric	Definition	ROI/Canal
Global TBR	θ/(β1 + β2)	mean z 19 canals
Frontal TBR	θ/(β1 + β2)	Fp1, Fp2, F3, F4, F7, F8, Fz
Prefrontal TBR	θ/(β1 + β2)	Fp1, Fp2, F3, F4
TBR midline	θ/(β1 + β2)	Fz, Cz
Frontal Alpha Asymmetry FAA	ln(α right) − ln(α left)	Fp2–Fp1 (±F4–F3)
Temporal beta stress	mean(β1 + β2)	T3, T4 (≈T7/T8)
Parietal EMG tension	β2	P3, P4, Pz

**Note**. θ = theta activity (4–7 Hz); β1 = low beta band (13–20 Hz); β2 = high beta band (20–30/35 Hz); α = alpha activity (8–12 Hz); ln = natural logarithm; mean = average signal value; TBR = Theta–Beta Ratio; FAA = Frontal Alpha Asymmetry; SMR = Sensorimotor Rhythm. Fp1, Fp2, F3, F4, F7, F8, Fz, Cz, T3/T4 (≈T7/T8), P3, P4, and Pz denote electrode locations according to the international 10–20 EEG system.

**Table 2:** Group comparisons for EEG markers.

Marker	M_K	SD_K	M_Z	SD_Z	T	*p*	d	p_adj
Frontal TBR	1.43	0.101	1.153	0.099	9.225	0.0	2.77	0.000 *
Global TBR	1.367	0.068	1.189	0.065	8.905	0.0	2.68	0.000 *
Prefrontal Theta Beta1	2.745	0.355	1.957	0.263	8.271	0.0	2.52	0.000 *
Motivation TBR midline	2.699	0.52	2.107	0.342	4.389	0.000	1.35	0.000 *
Temporal beta stress	19.774	2.224	17.435	2.204	3.52	0.001	1.06	0.002 *
Alpha Fp1	39.11	9.738	47.724	8.8	−3.077	0.004	−0.93	0.005 *
Parietal beta2 tension	15.824	2.74	13.721	2.043	2.856	0.007	0.87	0.009 *
Alpha Fp2	42.271	7.549	46.568	7.693	−1.882	0.067	−0.56	0.074
FAA ln	0.088	0.303	−0.021	0.24	1.309	0.199	0.4	0.199

M_K—Mean of the experimental group; SD_K of the experimental group. M_Z—Mean of the control group; SD_Z of the control group; * statistically significant results.

**Table 3:** Significant qEEG markers and their neurofunctional interpretation.

Significant qEEG Marker	Region/Area	Neurofunctional Meaning of the Area	Practical Implication
Frontal TBR	Fp1, Fp2, F3, F4, F7, F8, Fz	Frontal areas are involved in executive control, inhibition, planning, attention regulation, and behavioral monitoring.	Supports the use of structured learning strategies, clear task organization, short instructions, and executive-function support.
Global TBR	Mean from 19 EEG channels	Global activity reflects the general balance between slower and faster cortical rhythms across the scalp.	Suggests the need for general support of attention, learning readiness, and self-regulation across school activities.
Prefrontal Theta/Beta1	Fp1, Fp2, F3, F4	Prefrontal regions are associated with cognitive control, self-monitoring, working memory, emotional regulation, and goal-directed behavior.	Supports interventions focused on planning, working memory, emotional self-regulation, and predictable learning routines.
Midline TBR	Fz, Cz	Midline frontal-central regions are associated with sustained attention, cognitive effort, motivational engagement, and monitoring of ongoing activity.	Supports frequent feedback, task segmentation, motivational scaffolding, and monitoring of attention during learning.
Temporal beta stress	T3, T4	Temporal regions are involved in auditory–language processing, emotional reactivity, and integration of socio-emotional stimuli; increased beta may also reflect heightened arousal or muscle tension.	Supports stress-reduction strategies, emotionally safe classroom conditions, predictable routines, and regulation breaks.
Parietal beta2 tension	P3, P4, Pz	Parietal regions are involved in sensory integration, body-related processing, spatial attention, and attentional orientation; beta2 activity may partly reflect somatic or muscular tension.	Supports incorporation of relaxation, movement breaks, body-awareness strategies, and reduction of sensory overload.
Alpha Fp1	Left prefrontal area	The left prefrontal region is associated with approach-related motivation, emotional regulation, and cognitive engagement. Lower alpha may indicate altered frontal activation.	Suggests the need to monitor emotional regulation, motivation, and readiness to engage in learning tasks.

**Note.** The table includes only qEEG markers that significantly differentiated children from alcohol-affected families from controls after correction for multiple comparisons. The interpretations should be treated as neurofunctional correlates and not as direct indicators of functional impairment.

## Data Availability

Due to the nature of the health-related data, additional information is available by contacting the authors of the article directly.
